# Recordings in an integrating central neuron provide a quick way for identifying appropriate anaesthetic use in fish

**DOI:** 10.1038/s41598-018-36130-8

**Published:** 2018-12-03

**Authors:** Peter Machnik, Elisabeth Schirmer, Laura Glück, Stefan Schuster

**Affiliations:** Department of Animal Physiology, University of Bayreuth, D-95440 Bayreuth, Germany

## Abstract

In animal husbandry, livestock industry and research facilities, anaesthetic agents are frequently used to moderate stressful intervention. For mammals and birds, procedures have been established to fine-tune anaesthesia according to the intervention. In ectothermic vertebrates, however, and despite changes in legislation and growing evidence on their cognitive abilities, the presently available information is insufficient to make similarly informed decisions. Here we suggest a straightforward way for rapidly filling this gap. By recording from a command neuron in the brain of fish whose crucial role requires it to integrate and process information from all sensory systems and to relay it to motor output pathways, the specific effects of candidate anaesthesia on central processing of sensory information can directly and efficiently be probed. Our approach allows a rapid and reliable way of deciding if and at which concentration a given anaesthetic affects the central nervous system and sensory processing. We employ our method to four anaesthetics commonly used in fish and demonstrate that our method quickly and with small numbers of animals provides the critical data for informed decisions on anaesthetic use.

## Introduction

In many countries, legislation no longer distinguishes between ‘higher’ and ‘lower’ vertebrates, but requires for all vertebrates that anaesthetics are used in all interventions that could be stressful^1–5^. However, anaesthetic agents differ largely in their anaesthetising and side effects. Hence, even with equal legal treatment of all vertebrates, detailed evidence is needed to select the appropriate anaesthesia for a particular intervention. Such information is available in birds and mammals. Based on the sensory system or structures of the central nervous system on which they act, a carefully designed mix of anaesthetic agents can be chosen for any given intervention, taking even potential side effects into account^6^. For fish and other ectothermic vertebrates, however, we are presently lacking information about the effects of potential anaesthetic agents on sensory systems and sensory processing. This massively limits the possibilities to make similar informed decisions on anaesthesia so that there currently is a serious gap between legislative demands and the data required to fulfil them. Time is pressing, because anaesthetisation, particularly of fish, is extensively used, both in research facilities, where the zebrafish *Danio rerio* has become one of the most potent and widely used vertebrate model systems^7–9^, but also in the economically extremely important aquaculture industry^10^. According to the FAO, fish production increases year after year, reaching an annual volume of more than 170 million tonnes now^11^. With the continuous increase of fish production, anaesthetic quantities used in fish and the need of effective anaesthesia also grows continuously. Worldwide numbers are not available, but, for instance, in Norway state authorities track the used quantities of pharmaceuticals applied to fish. Despite responsible use, numbers indicate an exponential increase of anaesthetic quantities in aquaculture industry^12^. Given the steadily accumulating evidence on higher cognitive functions in fish^13–19^, given that legislation already demands it in a growing number of states and given the time needed for drug companies and legislation to establish new anaesthetics, it is clear that we do not have equally long time as we took in mammals and birds to establish appropriate data for ectothermic vertebrates that can be used in legal decision making^20,21^. It is therefore important to establish ways in which useful and yet reliable information can be obtained quickly both on potential novel anaesthetics and on the anaesthetics that are presently in use. Apart from their potential to make handling easier and to reduce stress, the effect of various anaesthetics on specific sensory systems particularly needs to be known for various concentrations to facilitate their aimed application for reducing suffering most efficiently.

**Figure 1:**
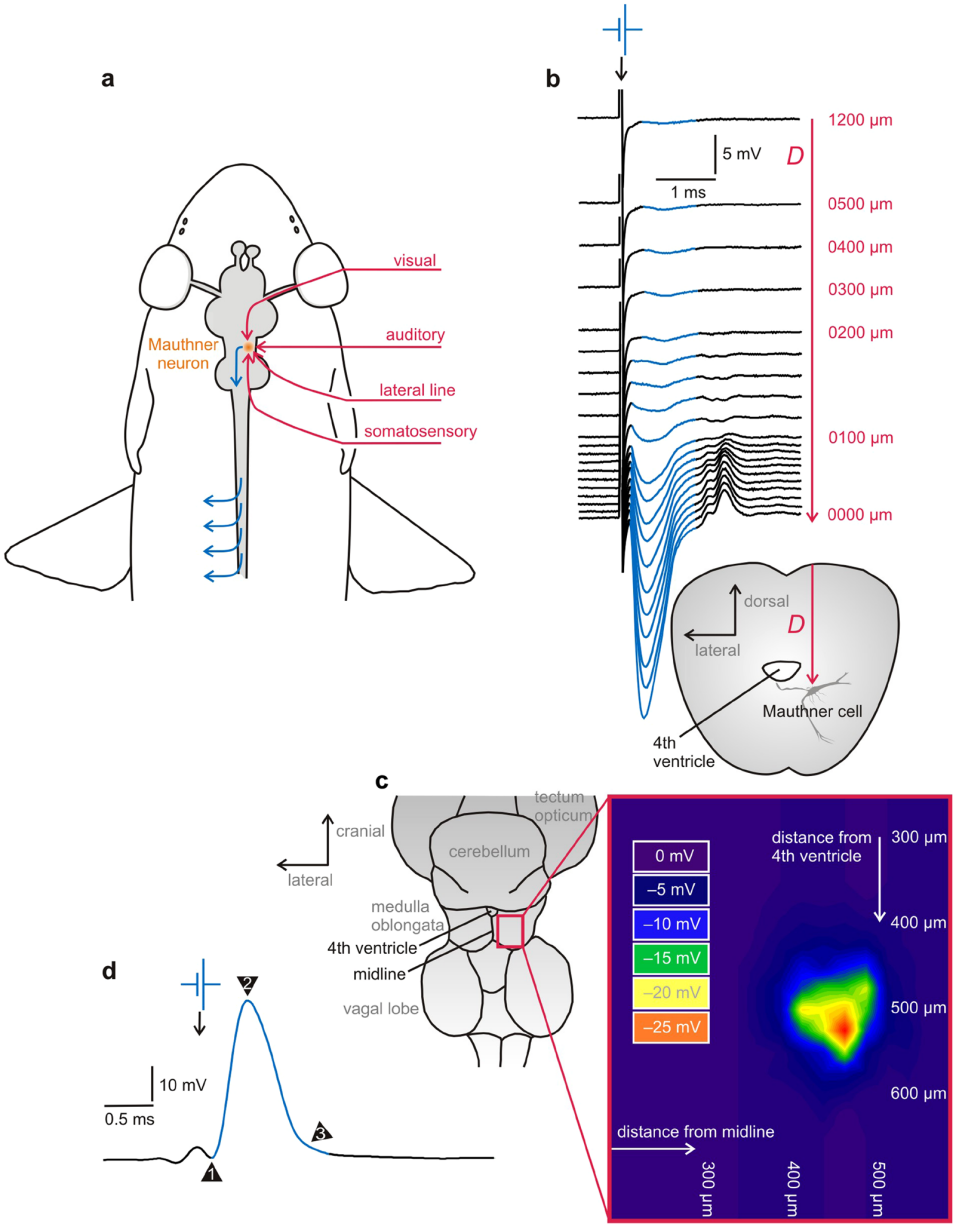
Brief overview of major features that make the Mauthner cell an interesting experimental system to elucidate the differential actions of anaesthetics. (**a**) Multisensory integration and motor output: Sketch of a teleost fish with central nervous system (grey), the right one of its two Mauthner neurons (orange spot) in the hindbrain, sensory input to the Mauthner neuron (red) and its motor output (blue). When input is suprathreshold, one of the two Mauthner neurons fires one action potential and this will cause body bending by activating trunk muscles on the contralateral side. (**b**–**d**) The Mauthner neuron is an identified neuron that is easy to find and to record from. Though buried deeply in the hindbrain, the Mauthner cell soma can be found for *in vivo* recording on the basis of an all-or-none field potential that emerges from an associated structure in direct vicinity, the axon cap, when the Mauthner axon is activated by stimuli applied to the spinal cord. (**b**) To illustrate this important feature, the known increase of this field potential during a direct approach from the medullary surface to the centre of the axon cap is shown for goldfish. *D* indicates the distance between the recording electrode and the centre of the axon cap at respective measuring position. (**c**) A map of the field potential amplitude at the depth of the goldfish Mauthner cell – about 1.2 mm under the surface of the medulla, with distance from major medullary landmarks (4th ventricle and midline) indicated (208 sampling points; right hemisphere; distance between points between 25 and 100 µm depending on steepness of change in field potential). (**d**) At the field potential maximum, advancing the electrode slightly further will allow recording from the Mauthner neuron. Its identity can be confirmed by several unique characteristics of its action potential, such as its short latency after spinal cord stimuli (1; arrowhead) and the absence of both overshoot (2) and undershoot (3).

Here we demonstrate that recording from the so-called Mauthner neurons, a pair of large identified neurons in the hindbrain of fish (and some amphibians)^22^ is ideally suited to address this challenging issue. The key is that their natural function requires these neurons to integrate information from all sensory systems and to rapidly issue a motor command that would allow the fish to rapidly escape from potential danger (Fig. 1a). We show that this system is ideally suited to determine quickly the effect of a given anaesthetic on various sensory systems, on central processing and motor output. Here we employed this system to provide a first suggestion of the use of four anaesthetics that could effectively be applied in fish and potentially some other ectothermic vertebrates. Two of them are benzocaine and the benzocaine derivative MS-222, which is currently the most commonly used anaesthetic in ectothermic animals^2,21,23,24^. The other two are 2-phenoxyethanol (2-PE) and Aqui-S, with the latter one widely used in aquaculture facilities^20^. Our findings thereby can be used as a first guide to scientists, veterinarians and aquaculture specialists until further pursuing our approach leads, in the coming years, to a finer picture with more options.

## Results

### Assaying the effect of anaesthetics on neuronal functionality

**Figure 2:**
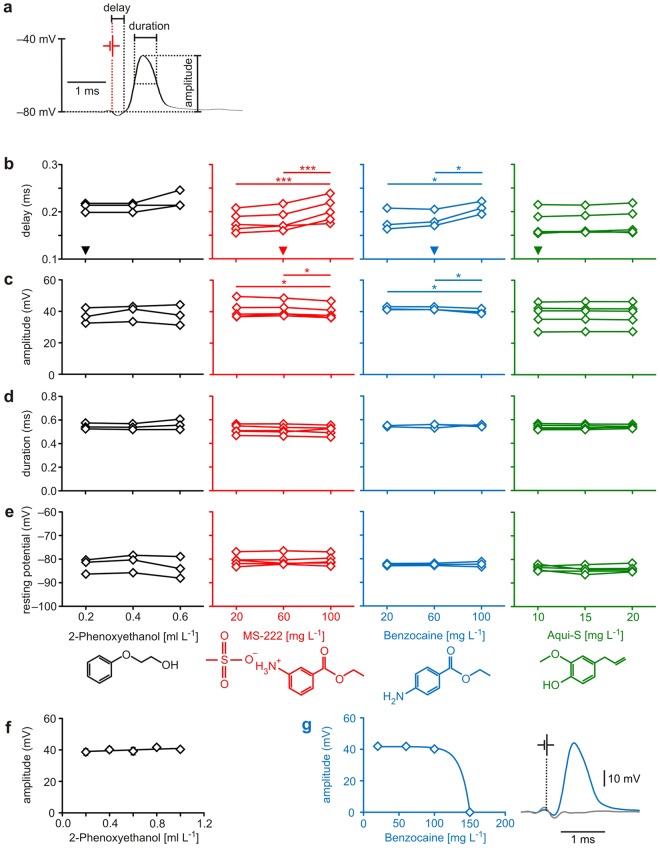
The anaesthetics benzocaine, MS-222, 2-PE and Aqui-S act differently on the Mauthner cell. (**a**) To assess potential differences in how the anaesthetics could affect the functionality of neurons in the central nervous system of fish, we determined the resting potential of the Mauthner neuron and properties of its action potential. (**b**) Both benzocaine (blue) and its derivate MS-222 (red) reduced conduction speed (i.e. increased delay) in a concentration-dependent fashion. Arrowheads in the graphs indicate the anaesthetic concentration needed for achieving surgical anaesthesia, respectively, referring to Neiffer and Stamper^28^. (**c**) At concentrations above 60 mg L^−1^ benzocaine and MS-222 caused a reduction in the amplitude of the action potential. (**d**,**e**) For concentrations up to 100 mg L^−1^ benzocaine and MS-222 did not affect the resting potential and the duration of the action potential. However, increasing benzocaine concentration above 100 mg L^−1^ blocked the functionality of the neuron (**g**). In 3 of 3 animals tested, no more action potentials were fired at the concentration of 150 mg L^−1^, as illustrated by recordings taken in the same fish at benzocaine concentrations of 20 mg L^−1^ (blue) and of 150 mg L^−1^ (grey). In contrast, the two anaesthetics 2-PE and Aqui-S neither affected the resting potential of the Mauthner neuron (**e**) nor its action potential (**b**–**d**). (**f**) Even increasing 2-PE concentration to 1 ml L^−1^ (5 times the concentration needed for establishing surgical anaesthesia) did not affect functionality. MS-222 and Aqui-S: *N* = 5 fish each; 2-PE and benzocaine: *N* = 3 fish each; * indicates *P* < 0.05; *** indicates *P* ≤ 0.001; significant differences between groups are indicated by horizontal lines with the level of significance indicated by asterisk(s).

The Mauthner neuron can easily be localised (Fig. 1b,c), identified (Fig. 1d) and accessed *in vivo* for intracellular recording using well-established techniques and criteria^25^. After having placed an electrode for recording the membrane potential of one of the two Mauthner neurons, examining the impact of anaesthetic agents on the animal’s central processing can be started. We first tested the effect of our selection of agents, applied in reasonably administrable concentration (see Methods), on neuronal functionality. For that we activated the Mauthner neuron by stimulating the spinal cord electrically (Fig. 1d). This allows to easily assay both the resting potential and characteristics of the action potential (i.e. its delay, amplitude, half-maximal duration) as measures for how anaesthetics act at the cellular level in neurons (Fig. 2a). We first applied anaesthetic concentrations commonly used in teleost fish: 0.2 to 0.6 ml L^−1^ 2-PE, 20 to 100 mg L^−1^ MS-222 and benzocaine and 10 to 20 mg L^−1^ Aqui-S, respectively^4,21,23,26–29^. To determine the anaesthetic impact of each agent, we ran concentration effect curves by changing the anaesthetic concentration while recording from the Mauthner cell. However, none of the applied concentrations of 2-PE or Aqui-S significantly affected any cellular property of the Mauthner neuron (Fig. 2b–e; repeated measures ANOVA: *r*^2^ ≤ 0.48, *P* ≥ 0.07 in all plots). In contrast, the two benzocaine derivates (MS-222 and benzocaine) increased the delay from spinal cord stimulation to the action potential in the Mauthner neuron in a concentration-dependent way (repeated measures ANOVA: *r*^2^ ≥ 0.89, *P* ≤ 0.01), and decreased the amplitude of the action potential (repeated measures ANOVA: *r*^2^ ≥ 0.69, *P* ≤ 0.01). The resting potential and the half-maximal duration of the action potential were not affected by MS-222 or by benzocaine anaesthesia (repeated measures ANOVA: *r*^2^ ≤ 0.16, *P* ≥ 0.51). Furthermore, we found no significant difference between the effects detected in the animals anaesthetised with MS-222 and those in the animals anaesthetised with benzocaine (paired *t* test: *P* ≥ 0.18). This indicates that both benzocaine derivates similarly affected neuronal properties.

Next, we asked whether the differences in how the anaesthetics affected cellular properties were simply due to different effective concentration levels or indicate inherent differences between the agents. We therefore increased the concentration further for 2-PE and for benzocaine, applying concentrations up to 1.0 ml L^−1^ of 2-PE and up to 150 mg L^−1^ of benzocaine. Both concentrations are substantially higher than needed for establishing surgical anaesthesia^20,28^. The findings fully confirmed that 2-PE does not affect cellular properties of the Mauthner neuron even at high concentrations. This is shown exemplarily for the amplitude of the action potential in Fig. 2f (linear regression analysis: *r*^2^ = 0.03, *P* = 0.37), but also held for all other measures (delay, half-maximal duration, resting potential; linear regression analysis: *r*^2^ ≤ 0.06, *P* ≥ 0.23 in all plots). In contrast, benzocaine at a concentration of 150 mg L^−1^ (i.e. at 2.5 times the surgical concentration^28^) terminated the capacity of the neuron to fire an action potential in 3 of 3 animals tested (Fig. 2g). Benzocaine and 2-PE thus provide examples of anaesthetics that either do not affect the functionality of neurons (2-PE) or reduce it in a concentration-dependent fashion (benzocaine) and with an effect already seen at concentrations applied in surgery or handling.

### Assaying the effects on hearing and acoustic processing

**Figure 3:**
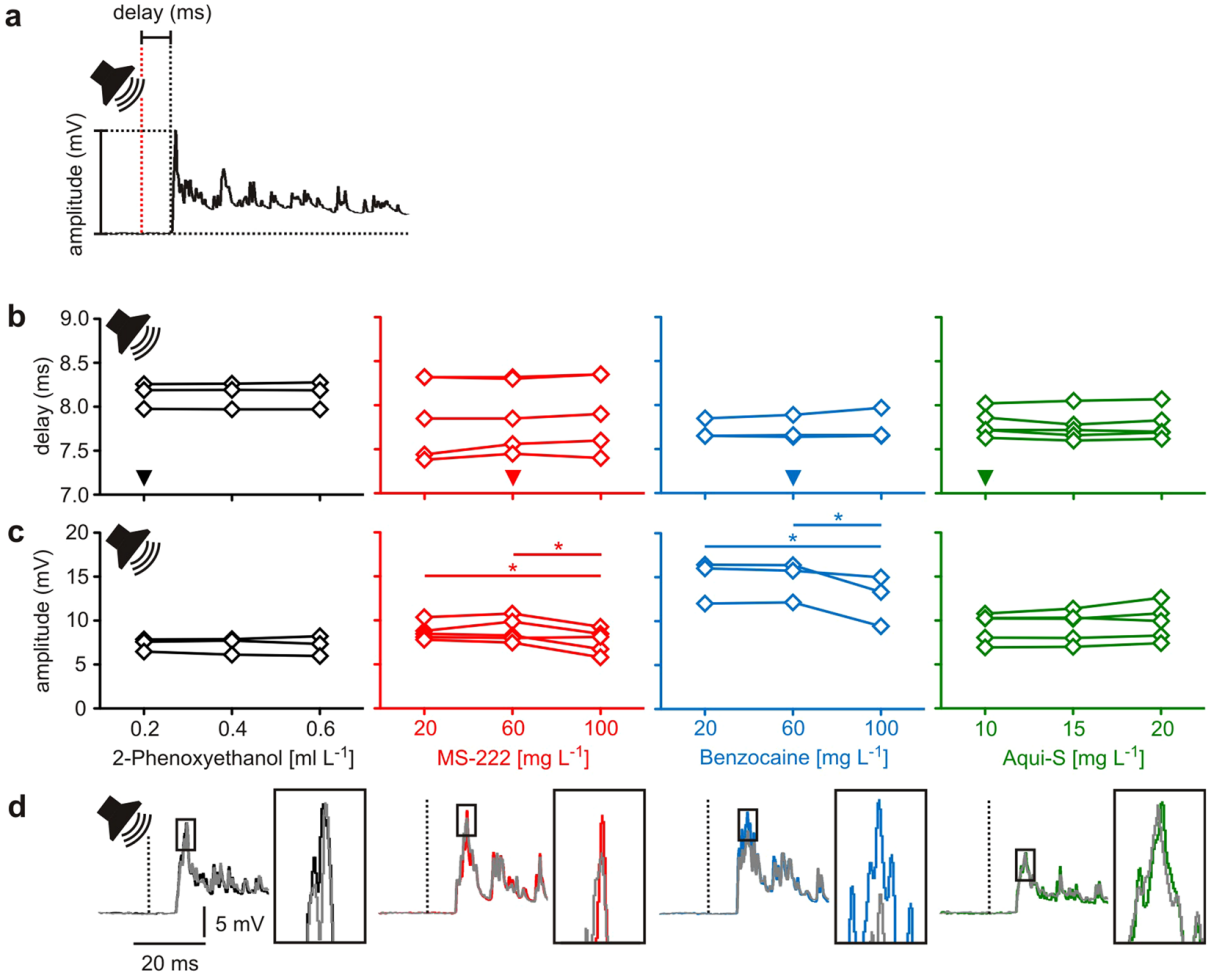
The effect of anaesthetics on hearing and auditory processing. (**a**) Acoustic stimulation elicits postsynaptic potentials (PSPs) in the Mauthner neuron, whose delay and amplitude provide an easy way to examine the effect of anaesthetics on hearing and auditory processing. (**b**,**c**) 2-PE and Aqui-S had no effect on delay and amplitude of the acoustically induced PSPs. However, MS-222 and benzocaine, when applied at concentrations above the surgical level of 60 mg L^−1^, significantly reduced the amplitude of acoustically induced PSPs. MS-222 and Aqui-S: *N* = 5 each; 2-PE and benzocaine: *N* = 3 each. * indicates *P* < 0.05. Significant differences between groups indicated by horizontal lines. The respective anaesthetic concentration needed for achieving surgical anaesthesia is indicated in the graphs of (**b**) by an arrowhead. (**d**) Representative examples of PSPs measured in the same animal under anaesthesia with the lowest (coloured PSP) and the highest concentration (grey PSP) used.

Both benzocaine and MS-222 reduced the amplitude of acoustically induced PSPs in a concentration-dependent fashion (Fig. 3c; repeated measures ANOVA: *r*^2^ ≥ 0.66, *P* ≤ 0.01). However, even high concentrations did not completely block acoustic PSPs (Fig. 3c). In the animals anaesthetised with 20 mg L^−1^ MS-222, the amplitude of acoustically induced PSPs was 8.7 ± 0.4 mV. Increasing the MS-222 concentration to 100 mg L^−1^, only decreased the amplitude to 7.7 ± 0.6 mV. In animals anaesthetised with benzocaine, the amplitude of acoustically induced PSPs was 14.8 ± 1.4 mV for 20 mg L^−1^ benzocaine and 12.9 ± 1.9 mV for 100 mg L^−1^. Despite the substantial increase in concentration both anaesthetics only moderately reduced PSP amplitude (by less than 15%). Even when benzocaine was applied at the concentration that prevented the firing of action potentials (150 mg L^−1^; Fig. 2g), it did surprisingly not block the acoustically induced PSPs in the Mauthner neuron, but only reduced its amplitude to 9.8 ± 0.8 mV. This is still 66% of the amplitude measured under anaesthesia established by applying only 20 mg L^−1^ benzocaine. While MS-222 and benzocaine thus had only a mild effect on the amplitude, they even had no detectable effect at all on the delay of the acoustically induced PSPs (Fig. 3b; repeated measures ANOVA: *r*^2^ ≤ 0.42, *P* ≥ 0.11).

Also both 2-PE and Aqui-S did not affect delay and amplitude of acoustically induced PSPs (Fig. 3b,c; repeated measures ANOVA: *r*^2^ ≤ 0.38, *P* ≥ 0.14). PSPs measured under differently deep anaesthesia are shown in Fig. 3d. In summary, none of the four anaesthetics blocked hearing and auditory processing even at very high concentrations.

### Assaying the effects on vision and visual processing

**Figure 4:**
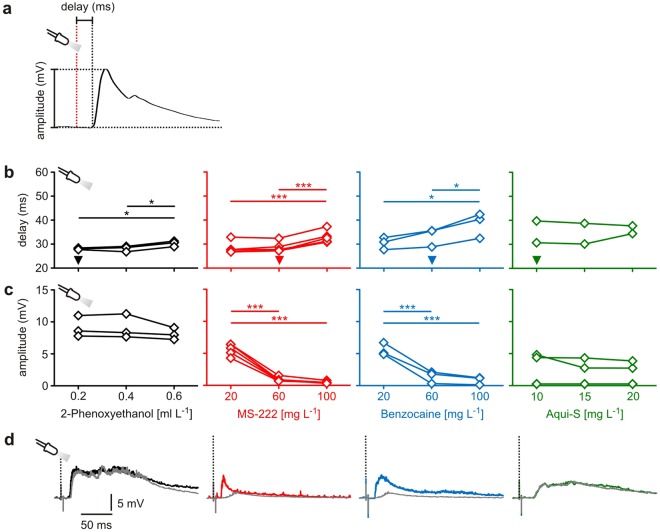
The effect of anaesthetics on vision and visual processing. (**a**) Even simple visual stimuli, such as light flashes, elicit postsynaptic potentials (PSPs) in the Mauthner neuron. Their delay and amplitude provide a convenient measure of how anaesthetics affect vision and visual processing. (**b**,**c**) 2-PE did not reduce the amplitude of visually induced PSPs (repeated measures ANOVA: *r*^2^ = 0.63, *P* = 0.13), but concentrations above 0.4 ml L^−1^ (i.e. concentrations 2 to 3 times above surgical concentration level) slightly increased their delay (repeated measures ANOVA: *r*^2^ = 0.90, *P* = 0.009). In contrast, MS-222 and benzocaine strongly affected PSP amplitude and delay and PSPs were almost undetectable above the surgical concentration of 60 mg L^−1^. In 2 of 5 experimental animals any Aqui-S concentration showed no effect on the visually induced PSPs, whereas in 3 further experimental animals Aqui-S reduced the amplitude of visually induced PSPs to zero. MS-222 and Aqui-S: *N* = 5 fish each; 2-PE and benzocaine: *N* = 3 fish each. * indicates *P* < 0.05; ** indicates *P* ≤ 0.01; ***indicates *P* ≤ 0.001. Significant differences between groups indicated by horizontal lines. Arrowheads indicate the anaesthetic concentration needed for achieving surgical anaesthesia in the graphs of (**b**), respectively, for better orientation. (**d**) Representative examples of PSPs measured in the same animal under anaesthesia with the lowest (coloured PSP) and the highest concentration (grey PSP) tested in the experiments.

Recording PSPs in the Mauthner neuron in response to visual stimuli allows to assay the effects of the four anaesthetics on vision and visual processing. Our measurements clearly show that benzocaine and its widely used derivate MS-222 block vision and/or visual processing in a concentration-dependent fashion (Fig. 4d). Figure 4b shows the effect of these agents on the delay of the light flash-induced PSPs in the Mauthner neuron (repeated measures ANOVA: *r*^2^ ≥ 0.89, *P* ≤ 0.01). Measured in the same animals, delay rose from 28.4 ± 1.1 ms under anaesthesia established by the application of 20 mg L^−1^ MS-222 to 33.0 ± 1.2 ms when anaesthetic concentration was increased to 100 mg L^−1^. In animals anaesthetised with benzocaine, delay was 30.5 ± 1.5 ms after the application of 20 mg L^−1^ benzocaine and 38.4 ± 3.0 ms for the concentration of 100 mg L^−1^. The amplitude of the visually induced PSP was affected even more impressively. It decreased drastically with increasing levels of both MS-222 and benzocaine (Fig. 4c; repeated measures ANOVA: *r*^2^ ≥ 0.98, *P* ≤ 0.0004). Increasing the anaesthetic concentration from 20 mg L^−1^ to 60 mg L^−1^ decreased the amplitude of visually induced PSP substantially, from 5.6 ± 0.4 mV to less than 1.0 mV. 100 mg L^−1^ further reduced the amplitude to less than 0.5 mV. In other words, animals anaesthetised with benzocaine or the benzocaine derivate MS-222 perform as if they were virtually blind for concentrations ≥60 mg L^−1^. This is compatible with reports on the impact of MS-222 on retinal function taken from *in vitro* measurements^30–32^. We would like to stress that our approach not only readily detects the effect, but also allows to conclude that MS-222 acts specifically on sensory function. The latter follows from a comparison of the effect of MS-222 on the auditory and the visually evoked responses in the Mauthner neuron. The absence of a correlated effect on both types of PSPs (correlation analysis on data of Figs 3c and 4c: *P* = 0.46) suggests that the effect is largely due to its effect on vision and not on central processing. This also held true for the effect of benzocaine.

In contrast to MS-222 and benzocaine, 2-PE did not block visually induced PSPs (Fig. 4d). The application of 2-PE only slightly increased the delay of visually induced PSPs for concentrations higher than 0.4 ml L^−1^ (Fig. 4b; repeated measures ANOVA: *r*^2^ = 0.90, *P* = 0.009), and did not affect the PSP amplitude (Fig. 4c; repeated measures ANOVA: *r*^2^ = 0.63, *P* = 0.13) at surgical concentrations. Also, the widely used anaesthetic Aqui-S does not always block vision. However, its effect on vision was remarkably variable (Fig. 4c). In 2 of the 5 experimental animals vision was fully intact at surgical levels. In contrast, Aqui-S, applied at the same concentration, effectively blocked visual PSPs in 3 of the 5 experimental animals (PSP amplitude < 0.5 mV). Note that all animals were checked before experiments (see Methods) for sensory induced responses and were clearly not blind. Furthermore, experiments were interspersed with experiments in which 2-PE was used for anaesthetisation and in which vision was unaffected, so that the variation in the effects could not be attributed to any parameters that might have changed in the setup between measurements in which Aqui-S had a strong effect and in which it had no effect on the visually induced PSPs. Aqui-S therefore appears to be highly variable in its effect on vision.

### Our approach allows significant statements based on small samples

**Figure 5:**
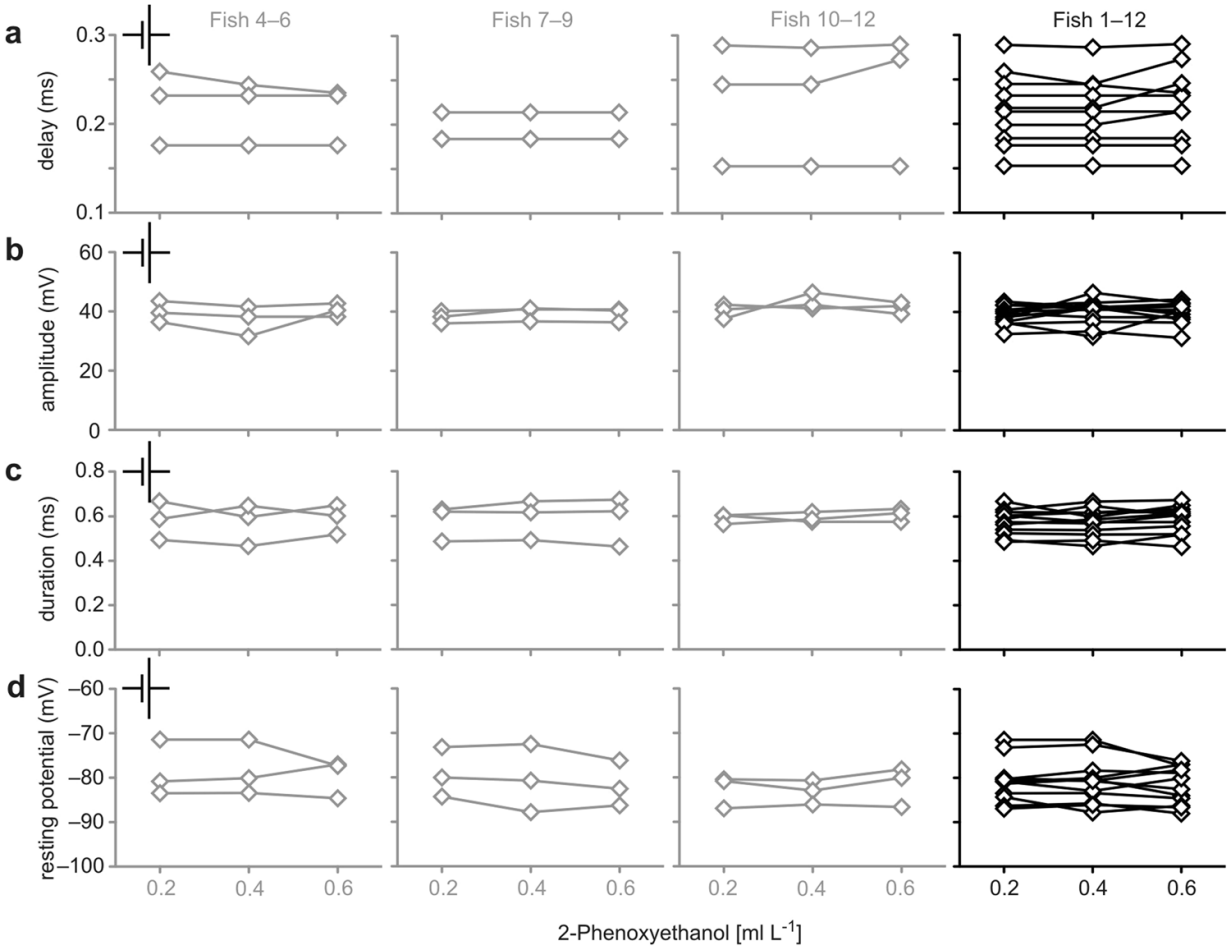
Evidence suggesting that small numbers of animals are sufficient to characterise effects of anaesthetics on neuronal functionality.  (**a–d**) shows data such as presented for *N* = 3 fish in Fig. 2b–e, but for three additional groups, also of *N* = 3 fish each, the far right column shows the results obtained when the *N* = 12 fish had been pooled. 2-PE was used as anaesthetic.

**Figure 6:**
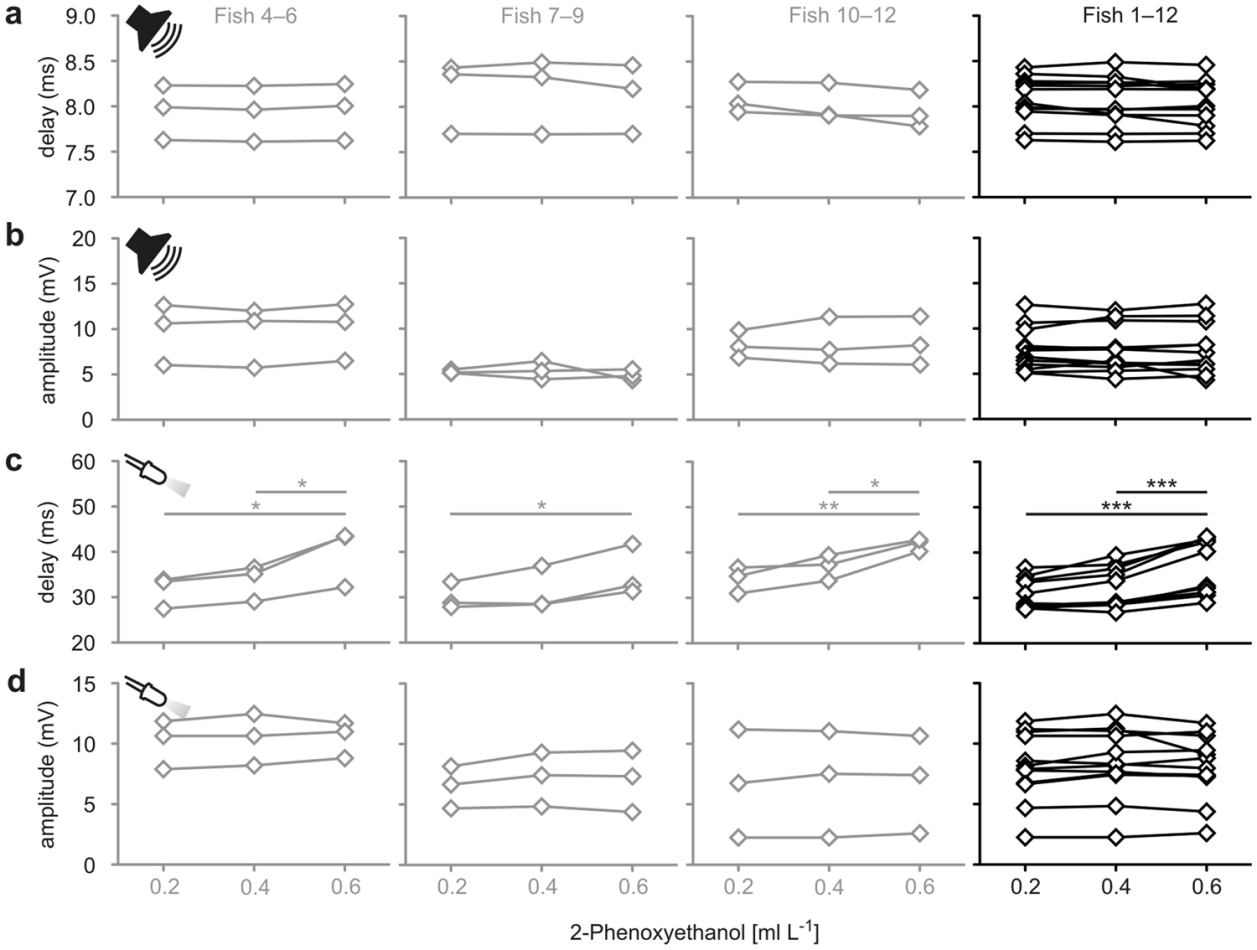
Evidence suggesting that small numbers of animals are sufficient to determine the effect of anaesthetics on sensory function. Analyses as in Figs 3 and 4, but with three additional groups, each of *N* = 3 fish. 2-PE was used as anaesthetic.

As a critical check of the practical usefulness of our approach we asked how many experimental animals are required to meaningfully assess the effect of an agent or pharmaceutical. To critically asses this decisive question, we took measurements in three additional groups of three animals each. The additional three groups were tested for the effect of 2-PE for anaesthetisation at concentrations in the range from 0.2 to 0.6 ml L^−1^ as described above (Figs 2b–e, 3b,c and 4b,c), so that we had four groups with a total of *N* = 12 experimental animals. To directly assess the variations between the groups of three animals, Figs 5 and 6 show the results of a characterisation of the three additional groups. We compared the conclusions made in the small-sample groups among each other, but also with the conclusions basing on the measurements of the pooled group (*N* = 12). It is striking that none of the small-sample groups gave an effect that differed from that obtained for the large group. In none of the small sample groups or the pooled group we detected a direct effect of 2-PE on the Mauthner neuron (repeated measures ANOVA: *r*^2^ ≤ 0.63, *P* ≥ 0.14 in all plots). Similarly, in no group did we detect an effect of 2-PE on the delay of acoustically induced PSPs and on the amplitude of both acoustically and visually induced PSPs (repeated measures ANOVA: *r*^2^ ≤ 0.71, *P* ≥ 0.09 in all plots). Furthermore, all four small-sample groups revealed the concentration effect of 2-PE on the delay of the visually induced PSPs (repeated measures ANOVA: *r*^2^ ≥ 0.86, *P* ≤ 0.02) just as in the pooled group (repeated measures ANOVA: *r*^2^ = 0.78, *P* < 0.001).

## Discussion

The goal of the current study was to explore how useful recordings of anti- and orthodromically stimulated Mauthner neurons would be to quickly obtain urgently needed reliable information on the various effects anaesthesia can have on sensory and neuronal function in fish. We demonstrate the power of our approach by studying the effect of four anaesthetics that are commonly used in ectothermic vertebrates: MS-222, benzocaine, 2-phenoxyethanol and Aqui-S. By monitoring neuronal functionality and visual and acoustic inputs to these command neurons^22,33–35^, we showed that recording in the Mauthner neuron allows to not only detect differential effects of the anaesthetics, but even successfully narrows down its site of action. Our approach allows a quick way of determining, which concentrations are needed for the desired effect. We show that small numbers of animals are sufficient and so our method is likely to quickly widen the spectrum of anaesthetics for fish and potentially other ‘lower’ vertebrates for the required more targeted application in experimentation, treatment and aquaculture. Moreover, our findings already provide a basis for a recommendation which of the anaesthetics to use for different purposes and at which concentration (Table 1).

**Table 1:** Practical guidelines for anaesthetic use based on our findings in goldfish.

Anaesthetic agent	Surgical conc.*	Functionality of CNS neurons		Handling in the presence of		Scientific study of	
		Affected	Vanished	Noise	Light	Hearing	Vision
2-PE (ml L^−1^)	0.2	no	no	no	no	yes	<0.6
MS-222 (mg L^−1^)	60	>60	n.d.	no	≥60	<100	no
Benzocaine (mg L^−1^)	60	>60	>100	no	≥60	<100	no
Aqui-S (mg L^−1^)	10	no	no	no	no	yes	no

*Required concentration according to Neiffer and Stamper^28^; n.d. = not determined.

Our approach exploits the function and accessibility of a pair of identified neurons in the hindbrain of most fish species and in many amphibians, that can be identified from one animal to the next. The so-called Mauthner neurons form the centre of a network, that is crucial to elicit a life-saving escape behaviour in response to a threat, such as a suddenly approaching predator. To achieve this, the Mauthner neurons integrate and process information forwarded from all of the animal’s sensory systems (Fig. 1a) to properly assess the necessity for driving an escape response^22^. This property makes the Mauthner neuron an ideal substrate for efficiently obtaining the differential effects agents have on central nervous processing and sensory function in fish. We show that important conclusions can already be obtained from small numbers of animals, a highly desirable property from an ethical point of view^36^ and needed to speed up data acquisition. Measurements taken from three fish were sufficient for determining the concentration dependence and general action of our sample of anaesthetics on neuronal and sensory function (Figs 5 and 6) and could not be improved by using larger samples of 12 fish. Even small effects could be reliably detected in the small groups of three fish, such as the concentration dependency of the delay of visually induced PSPs for 2-PE application (Fig. 4b).

Our findings strongly underline the importance of having detailed information available on anaesthetic effects in fish. We show that even the agents currently used to anaesthetise and to calm fish substantially differ in their effects. This is perhaps most striking in benzocaine and Aqui-S, two widely and indiscriminately used anaesthetics for fish. Even when benzocaine is given at a concentration that blocks firing of the Mauthner neuron, sound can still elicit sizeable PSPs in the same cell. Similarly, when benzocaine is given at a concentration that blocks visually induced PSPs, then activation of the neuron is still possible as well as sound induced PSPs. In the case of Aqui-S, an agent that is widely used in aquaculture facilities, we discovered that a remarkable degree of unpredictability exists selectively for visually induced responses, but not for acoustically induced responses and neuronal function. Hence, Aqui-S is a potent agent for handling and for reducing stress in fish, but not for blocking vision or for the scientific study of visual functions (Table 1). Our findings also underline the importance of using other anaesthetics besides benzocaine derivates like MS-222, the most commonly used anaesthetic in scientific work. It cannot be used, for instance, in studies on visual function, in which it should be substituted with 2-PE (Table 1). In studies on hearing all anaesthetics of our sample would be equally well suited. This means, however, also that none of them is capable of reducing strain of fish in particularly noisy environments. Presently our suggestions that we have condensed in Table 1 are based, of course, on experiments performed in goldfish. In absence of any other data, it would still be useful to operate on the basis of Table 1, even for other ectothermic vertebrates for which we lack any data. Of course, it is also possible to quickly widen the approach introduced here to other species and to use it to widen the spectrum of useful agents for targeted applications in fish.

In conclusion, we demonstrated here how recordings in the Mauthner neuron can quickly and systematically help filling the gap that currently exists between legislation and informed decision-making on anaesthesia in ectothermic vertebrates. By scanning the effects of further candidate agents, and by exploring their effects in a few more key species of fish, our approach will in the coming years contribute to achieving a reasonable and targeted anaesthesia in fish and will be of value for other ectothermic vertebrates, for which any information is presently lacking.

## Methods

### Experimental animals

We used *N* = 25 goldfish (*Carassius auratus* (Linnaeus, 1758), Cypriniformes) of either sex with standard lengths from 7 to 9 cm. The fish were obtained commercially from an authorised specialist retailer (Aquarium Glaser GmbH, Rodgau, Germany). Before used in an experiment, fish were kept for at least 12 weeks. In this period, they were maintained in a group at 20 °C and 12:12 h light/dark photoperiod in a tank (250 × 50 × 50 (cm)) filled with fresh water (water conductivity: 300 µS cm^−1^; pH 7.5; total hardness of water: 7.7°dH; NH_4_^+^ <10 µg L^−1^; NO_2_^−^ <5 µg L^−1^; NO_3_^−^ <5 mg L^−1^). Water of the same quality was used in the electrophysiological recording chamber. Fish were fed with common fish food (sera goldy; sera GmbH, Heinsberg, Germany) and defrosted red mosquito larvae. Animal care procedures, surgical procedures and experimental procedures were in accordance with all relevant guidelines and regulations of the German animal protection law and explicitly approved by state councils (Regierung von Unterfranken, Würzburg, Germany). Before selecting fish for an experiment, we checked that they respond to visual and acoustic stimuli: they had to show escape responses both to rapid hand movements in front of the aquarium and to knocking on the aquarium.

### Anaesthesia

We tested the effect of four anaesthetic agents commonly used in fish: (i) 2-phenoxyethanol (2-PE; 1-hydroxy-2-phenoxyethane; Sigma-Aldrich, Steinheim, Germany), (ii) ethyl-3-aminobenzoate methanesulfonate (also known as tricaine, TMS or MS-222; Sigma-Aldrich, Steinheim, Germany), (iii) benzocaine (Sigma-Aldrich, Steinheim, Germany; solved 1:10 in 95% ethanol), and (iv) Aqui-S (Scanvacc, Hvam, Norway). The use of one of these anaesthetics in a given experiment was selected at random to ensure that any differences could not be caused by unintended changes in the experimental setup or by undetected changes in the animals’ state. Each of the experimental fish was solely exposed to one of the anaesthetic agents to exclude potential interactions between the anaesthetics. Anaesthetic concentration levels, particularly surgical concentrations, were chosen based on appropriate references^4,21,23,26–29^. For the appropriate use of Aqui-S, an anaesthetic widely used in aquaculture facilities, we additionally used information provided by Aqui-S New Zealand Ltd (http://www.aqui-s.com/aqui-s-products/aqui-s) (2018). Concentration levels ranged from 0.2 to 1.0 ml L^−1^ for 2-PE, 20 to 100 mg L^−1^ for MS-222, 20 to 150 mg L^−1^ for benzocaine, and 10 to 20 mg L^−1^ for Aqui-S. 20 mg L^−1^ of the anaesthetics MS-222 and benzocaine cause slight anaesthetisation in fish (stage II anaesthesia^1,20^), whereas all other applied anaesthetic concentrations cause at least surgical anaesthetisation (stage III anaesthesia)^21^. Before starting any surgical intervention, fish were surgically anaesthetised (stage III anaesthesia^1,20^) by application of either 0.4 ml L^−1^ 2-PE, 60 mg L^−1^ MS-222 or benzocaine or 20 mg L^−1^ Aqui-S for 15 min. We generally confirmed the sufficiency of the anaesthetisation after total loss of equilibrium by carefully exerting pressure to the fish’s caudal peduncle. In responsive fish, this kind of touch reliably triggers an escape response and subsequent swimming behaviour. When this stimulation (and subsequent handling) yielded no response, then the fish was positioned in the electrophysiological recording chamber and artificial respiration was established with aerated water flowing via a tube through the mouth and out over the gills at a flow rate of 80 ml min^−1^. Respiration water thereby was delivered to the fish from a reservoir (respiration water tank) using a suitably adjusted pump (EHEIM universal 300; EHEIM GmbH & Co. KG, Deizisau, Germany; regular power: 300 L h^−1^, adjusted to 4.8 L h^−1^). To maintain anaesthesia, the respiration water always contained the same anaesthetic as used for establishing anaesthesia. We started experiments randomly either with the lowest concentration used in the respective experiment or with the highest one. To examine anaesthetic concentration effects, we then changed the concentration level within a particular animal, while simultaneously recording intracellularly from the Mauthner neuron. To increase the concentration level during the experiment, we added additional anaesthetic to the respiration water. To quickly establish a uniform mixture of respiration water and anaesthetic, we used a circulation pump (EHEIM universal 600; EHEIM GmbH & Co. KG, Deizisau, Germany; power: 600 L h^−1^) in the respiration water tank. To reduce the anaesthetic concentration, we added additional water of the same quality and temperature to the respiration water tank. After changing the anaesthetic concentration level, we always gave an acclimatisation period of 15 min before the next measurements were taken. This interval was chosen to be significantly beyond the estimated time (<6 min) needed by the used anaesthetics to impact on the animal’s physiology by simple add-on to the water surrounding the fish^23,27^.

### Surgical procedure

To access the Mauthner cells, we exposed the hindbrain by opening the skull from above using a bone rongeur. Additionally, we exposed a piece of the spinal column (about 5 mm length) in the region of the trunk from the side. The large axons of the Mauthner neurons run down the complete spinal cord and can be activated by applying electrical pulses to the spinal cord. Activation of both Mauthner cells causes typical twitching of the experimental animal. Note that none of the used anaesthetics applied in surgical concentration (Table 1) ceased the massive muscle activation after firing the Mauthner neurons. After testing the correct positioning of the homemade bipolar stimulation electrode forwarding electrical pulses to the spinal cord, we therefore had to immobilise the experimental animal for intracellular *in vivo* recording by injecting d-tubocurarine (1 µg g^−1^ body weight; Sigma-Aldrich, Steinheim, Germany).

After finishing recording, the experimental animal was sacrificed immediately and without recovery from anaesthesia by mechanically destroying the brain.

### Experimental procedure

We used a bridge-mode amplifier (BA-01X; npi electronic GmbH, Tamm, Germany) in current clamp mode for intracellular recordings with sharp electrodes. Recording electrodes (4–7 MΩ) were pulled from 3 mm-glass capillaries (G-3; Narishige Scientific Instrument Lab, Tokyo, Japan) by using a vertical electrode puller (PE-22; Narishige International Limited, London, UK) and filled with 5 M potassium acetate. A motorised micromanipulator (MP-285; Sutter Instrument, Novato, CA, USA) was used to position and to move the recording electrode. The reference electrode was positioned in muscle tissue. Recordings were filtered (Hum Bug Noise Eliminator; Quest Scientific, North Vancouver, BC, Canada) and digitised (A/D converter Micro1401; Cambridge Electronic Design Limited, Cambridge, UK) at 50 kHz. For further processing and analysis we used the acquisition software package Spike2 (version 6; Cambridge Electronic Design Limited, Cambridge, UK). After localisation and identification of one of the two Mauthner cells by using well-established techniques^22,25,37^, we determined the resting potential of the Mauthner cell, properties related to the Mauthner action potential (delay, action potential amplitude and its half-maximal duration) and properties related to acoustically and visually induced PSPs^37^, respectively. Delay was taken as the time from onset of the stimulus to the first deflection of the membrane potential away from resting potential. For amplitude we determined the difference between the resting potential and the maximum of the action potential. To elicit an action potential in the Mauthner cell, we stimulated the spinal cord electrically (pulse amplitude: up to 65 V, as required, but regularly between 8 and 12 V; pulse duration: 10 µs; stimulation rate: 2 Hz). Electric pulses thereby were delivered by a constant-voltage isolated stimulator (DS2A2 – Mk.II; Digitimer Ltd., Hertfordshire, UK). For acoustic stimulation, we used a multifunctional active loudspeaker (The box pro Achat 115 MA; Thomann GmbH, Burgebrach, Germany). The loudspeaker generated a short acoustical broadband pulse (duration 1 ms; frequency distribution from 25 to 1000 Hz; peak amplitude at 300 Hz) with a sound pressure level (SPL) of 145 dB *re* 1 µPa. We measured SPL under water at the position of the fish in the recording chamber with a hydrophone (Type 8106; Brüel & Kjær, Nærum, Denmark). Visual stimuli were 7 ms-light flashes delivered by a light emitting diode (LED) (RS Components GmbH, Mörenfelden-Walldorf, Germany) directly positioned in front of the ipsilateral eye. The LED peak radiation at about 569 nm was 700 µW m^−2^ nm^−1^ and the width at 100 µW m^−2^ nm^−1^ was 56 nm (range: 543 to 599 nm).

### Statistical analysis

Statistical tests were run by using the software package GraphPad Prism 5.0 f (GraphPad Software, Inc., La Jolla, CA, USA) and performed two-tailed with α = 0.05. We tested deviation from normal distribution using the Shapiro-Wilk test. We tested departure from linearity for 2-PE and benzocaine concentration level effects by using a runs test. If the runs test revealed no significant deviation from linearity, we performed a linear regression analysis, otherwise we searched for a better corresponding non-linear fit. To factor out interindividual differences, we changed anaesthetic concentration within particular experimental animals and then used repeated measures design for statistical analysis (e.g. paired *t* test, repeated measures ANOVA). To compare the impact of MS-222 and benzocaine on neuronal functionality, we also used repeated measures design. Here, we used anaesthetic concentration for pairing. Averages are given as mean ± standard error of mean (s.e.m.). The heat map (Fig. 1c) was constructed in SigmaPlot 11.0 (Systat, Inc., Erkrath, Germany). *n* labels the number of analysed values; the number of analysed experimental animals is labelled *N*.
